# On the Effects of Clothing Area Factor and Vapour Resistance on the Evaluation of Cold Environments via IREQ Model

**DOI:** 10.3390/ijerph22081188

**Published:** 2025-07-29

**Authors:** Francesca Romana d’Ambrosio Alfano, Kalev Kuklane, Boris Igor Palella, Giuseppe Riccio

**Affiliations:** 1 DIIN—Dipartimento di Ingegneria Industriale, Università di Salerno, Via Giovanni Paolo II 132, 84084 Fisciano, Italy; fdambrosio@unisa.it; 2 Dutch Academy for Crisis Management and Fire Service (NACB), Netherlands Institute for Public Safety (NIPV), P.O. Box 7112, NL-2701 AC Zoetermeer, The Netherlands; kalev.kuklane@nipv.nl; 3 Chair of Biosystems Engineering, Institute of Forestry and Engineering, Estonian University of Life Sciences, Fr. R. Kreutzwaldi 56, 51006 Tartu, Estonia; 4 DII—Dipartimento di Ingegneria Industriale, Università degli Studi di Napoli Federico II, Piazzale Vincenzo Tecchio 80, 80125 Naples, Italy; riccio@unina.it

**Keywords:** cold stress, IREQ, duration limit exposure, clothing area factor, vapour resistance, ISO 11079

## Abstract

The IREQ (Insulation REQuired) index is the only reliable and effective model for predicting and evaluating the protection given by a clothing ensemble in cold environments. Even with the growth of studies aimed at assessing the thermophysical characteristics of clothing, IREQ remained unaltered from Holmér’s original formulation four decades prior. This paper focuses on the effect of the evaluation of the clothing area factor and the resultant vapour resistance on the assessment of cold environments via IREQ. Obtained results reveal meaningful variations in the duration limit exposure (up to 5 h), whereas IREQ values remain unchanged. Observed phenomena could be interesting when discussing the revision of the ISO 11079 standard, which prescribes using IREQ for the determination and interpretation of cold stress.

## 1. Introduction

### 1.1. Background

As observed by Petersson et al. [1], despite the increased interest in heat stress due to global warming, in temperate climates, the mortality is still higher in winter than in summer [2,3]. Cold exposure is typical in some outdoor work environments, including open pit miners, oil and gas industry workers, foresters, soldiers, construction and road industry personnel and occurs indoors in several industrial activities (e.g., milk, fish, meat industry, food distribution and pharmaceutical units) [4,5,6]. The most common problems/illnesses associated with cold exposure include hypothermia, pain in the extremities up to frostbite, impaired mobility and operational capacity due to the weight of clothing, and reduced physical capacity of the body [4,7,8,9,10].

The increased interest in occupational safety and health gave rise to the update of the legislation [11] and the promotion of public awareness, education and investigation in this field and in the formulation of a three-phase strategy called SOBANE (Screening, OBservation, ANalysis, Expertise) [6,12,13], which inspired the ISO 15265 [14] and, specifically for cold environments, the ISO 15743 [15]. This standard reports models and methods for cold-risk assessment and management, a checklist for identifying cold-related problems at work, a methodology based on subjective analysis aimed at identifying those individuals with symptoms that increase their cold sensitivity, and guidelines on how to apply thermal standards and other validated scientific methods when assessing cold-related risks. As far as the evaluation of the cooling of the body as a whole, ISO 15743 recommends the evaluation of the Insulation Required IREQ [16,17,18], which is also used by ISO 15265 to assess the class of risk in cold environments both in the short and long term.

Forty years after its formulation, several investigations based on physiological, objective and subjective analyses demonstrate the reliability of the IREQ model [19,20,21,22,23], even if compared with other available models [24,25]. Most specifically, studies carried out during military operations reveal that clothes worn by soldiers exhibit thermal insulation values generally in agreement with IREQ or slightly higher, leading to a slight increase (1 °C) in the core temperature [19,20]. Other studies seem to confirm that the claim of operators exposed to cold conditions is typically due to behavioural issues: workers prefer to wear lighter clothes to increase the ease of movement [22,23] and often do not accurately capture the correlation between the cold protection provided by clothing and the environmental conditions [26].

Other criticisms about IREQ focused on a lack of a database of experimental investigations as the basis of its validation [27,28,29,30], the impossibility of considering short time exposure and variations in work intensity [31], and some inconsistencies in its calculation by software due to systematic errors [32]. In addition, the influence of wind action and body movements on clothing insulation and vapour resistance values relies on algorithms that differ from those reported in ISO 9920 [33,34].

Finally, recent experimental studies focused on the determination of the clothing area factor as a function of the basic clothing insulation have resulted in the formulation of new and more robust algorithms. These algorithms represent an advancement compared to those prescribed by existing standards in the field of physical environment ergonomics [35,36], but their influence on the IREQ formulation has yet to be investigated.

### 1.2. Aim of This Paper

Despite its limitations, the IREQ model remains the only tool for evaluating global and local cooling in cold environments. That is why it is used by ISO standard 11079, which deals with the assessment of cold environments in occupational health and industrial hygiene, and to combine weather forecasting with personal factors to offer guidance on the risks of daily cold exposures [1]. The model, unchanged since 1993, suffers from significant drawbacks. Its implementation is inconsistent, and its underlying rationale fails to reflect recent advancements in clothing thermophysical properties research and standardization. Notably, the model lacks the new algorithms for calculating clothing area factor and evaporative resistance, leaving these crucial aspects unaddressed.

The aim of this paper is to verify to what extent the evaluation of these two quantities influences the body heat storage rate and the exposure duration limit *D_lim_*. The results obtained will constitute solid arguments for revising the IREQ model and the current ISO standard 11079 and implementing more reliable software.

The next section aims to help readers understand the IREQ model’s rationale and its detailed application. Subsequently, it will address the open issues and relevant literature that motivate the present investigation.

## 2. The IREQ Model

### 2.1. Model Features

IREQ represents the clothing insulation value needed to maintain the body heat balance. It is calculated from the well-known heat balance equation on the human body [35].

(1) $$M-W=E_{\mathrm{res}}+C_{\mathrm{res}}+E+K+R+C+S$$

where

*C* convective heat flow, W m^−2^;

*C*_res_ respiratory convective heat flow, W m^−2^;

*E* evaporative heat flow at the skin, W m^−2^;

*E*_res_ respiratory evaporative heat flow, W m^−2^;

*K* conductive heat flow, W m^−2^;

*M* metabolic rate, W m^−2^;

*R* radiative heat flow, W m^−2^;

*S* body heat storage rate, W m^−2^;

*W* rate of mechanical power, W m^−2^.

By assuming negligible the contribution of the heat transfer by conduction (K = 0), under steady conditions (S = 0), Equation (1) can be arranged as

(2) $$R+C=M-W-E_{\mathrm{res}}-C_{\mathrm{res}}-E$$

Convective, radiative and evaporative heat flows can be calculated as follows:

(3) $$C=f_{\mathrm{cl}}h_{c}\left(t_{\mathrm{cl}}-t_{a}\right)$$

(4) $$R=f_{\mathrm{cl}}\varepsilon_{\mathrm{cl}}\frac{A_{r}}{A_{\mathrm{Du}}}\left[{\left(t_{\mathrm{cl}}+273\right)}^{4}-{\left(t_{r}+273\right)}^{4}\right]$$

(5) $$E=w\frac{p_{\mathrm{sk},s}-p_{a}}{R_{e,T,r}}$$

where

*A_r_* effective radiating area of a body, m^2^;

*A*_Du_ Du Bois body surface area, m^2^;

*f*_cl_ clothing area factor, ND;

*h*_c_ convective heat transfer coefficient, W m^−2^ K^−1^;

*p*_a_ water vapour partial pressure, kPa;

*p*_sk,s_ saturated water vapour pressure at skin temperature, kPa;

*t*_a_ air temperature, °C;

*t*_cl_ clothing surface temperature, °C;

*t*_r_ mean radiant temperature, °C;

*R_e,T,r_* resultant evaporative resistance of clothing and boundary air layer, m^2^ kPa W^−1^;

*w* skin wittedness, ND;

*ε*_cl_ emissivity of the clothing surface, ND.

On the basis of Equations (1) and (2), *IREQ* can be finally obtained by means of this equation:

(6) $$IREQ=\frac{t_{\mathrm{sk}}-t_{\mathrm{cl}}}{R+C}$$

Equation (6) contains two unknown variables: *IREQ* and the clothing surface temperature *t_cl_*; therefore, it has to be solved for *t_cl_* by means of an iterative numerical procedure (see Appendix A for related details).

As the primary goal of the IREQ model is to analyze whether the clothing ensemble worn by the subject provides enough insulation to sustain the heat balance with no storage (S = 0), the resultant clothing insulation *I_cl,r_* covering the body under the current situation should be calculated and then compared with *IREQ*. The calculation of *I_cl,r_* value is obtained by the resultant clothing insulation value and the resultant boundary layer thermal insulation according to Equations (7)–(10) as indicated by the ISO standards 9920 [34] and 11079 [18].

(7) $$I_{\mathrm{cl},r}=I_{T,r}-\frac{I_{a,r}}{f_{\mathrm{cl}}}$$

(8) $$I_{T,r}=I_{T}\left[0.54e^{\left(0.075\cdot \ln a_{p}-0.15v_{a}-0.22v_{w}\right)}-0.06\cdot \ln a_{p}+0.5\right]$$

(9) $$I_{a,r}=0.092e^{\left(-0.15v_{a}-0.22v_{w}\right)}-0.0045$$

(10) $$I_{T}=I_{\mathrm{cl}}+\frac{I_{a}}{f_{\mathrm{cl}}}$$

where

*a*_p_ air permeability of outer fabric, L s^−1^ m^−2^;

*I*_a_ static boundary layer thermal insulation, m^2^ K W^−1^;

*I*_a,r_ resultant boundary layer thermal insulation, m^2^ K W^−1^;

*I*_cl_ basic clothing insulation, m^2^ K W^−1^;

*I*_cl,r_ resultant clothing insulation, m^2^ K W^−1^;

*I*_T_ basic total insulation, m^2^ K W^−1^;

*I*_T,r_ resultant total insulation, m^2^ K W^−1^;

*v*_a_ air velocity, m s^−1^;

*v*_w_ walking speed, m s^−1^;

*I_a_* = 0.085 m^2^ K W^−1^.

The walking seed value, if unknown, can be calculated as follows [34]:

(11) $$v_{w}=0.0052\left(M-58.2\right)$$

Equations (8) and (9) apply for 0.4 m s^−1^ ≤ *v_a_* ≤ 18 m s^−1^ and 0 m s^−1^ ≤ *v_w_* ≤ 1.2 m s^−1^.

Regarding the IREQ interpretation criteria, there are two possibilities:

1. *IREQ* < *I_cl,r_*: there is no risk for cold. However, if IREQ << I_cl,r_, then there may be a risk of overheating and excess sweating, which may lead to discomfort and cold-related issues later.
2. *IREQ* > *I_cl,r_*: worker safety is not necessarily guaranteed. More specifically, if the body heat loss is within 144 kJ m^−2^, there is no risk to health, provided that the extremities are properly protected. However, if this limit for heat loss is exceeded, it is necessary to assess the maximum safe exposure time.

In situation (2), for low strain conditions, it may be sufficient to have thermal insulation that satisfies the balance, *IREQ_neu_*, or it may be necessary to have thermal insulation that guarantees safety even for high strain, *IREQ_min_*. In both cases, duration-limited exposure *D_lim_* should be calculated based on acceptable levels of body cooling:

(12) $$D_{\lim}=-\frac{Q_{\lim}}{S}$$

**Figure 1:**
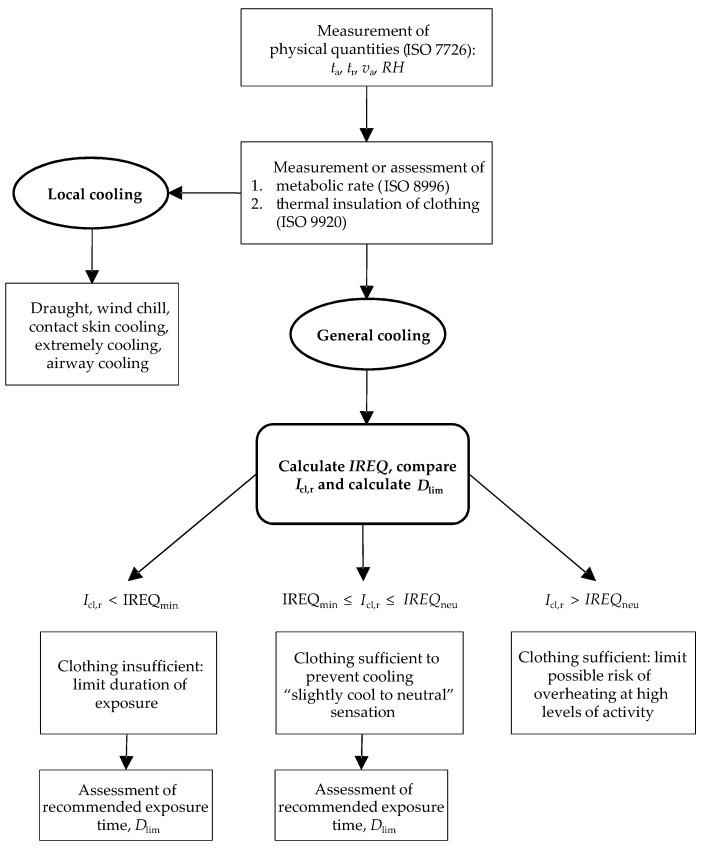
Procedure for calculating and interpreting the *IREQ* value according to ISO 11079 [18].

The heat storage rate *S* in Equation (12) can be obtained from Equations (1) (K = 0) and (6) (IREQ = I_cl,r_) by an iterative procedure. In Figure 1, the procedure for calculating and interpreting IREQ is shown, while suggested physiological criteria for the determination of *IREQ* and *D_lim_* are reported in Table 1.

### 2.2. Open Issues

Since 1993, knowledge in the field of cold thermal environments has advanced, but the only modification to the IREQ model has been the introduction of special algorithms for accounting for the effect of wind action and body movements on the basic clothing insulation values. In contrast, two fundamental aspects remain unexplored: the clothing area factor *f_cl_*, systematically investigated in [36], and the evaluation of the resultant (or dynamic) vapour resistance of clothing.

The clothing area factor *f_cl_* is defined as the ratio of the outer surface area of the clothed body to the surface area of the body. Its evaluation is a crucial step of the IREQ model because it affects convective, radiative, and evaporative heat losses, and total clothing insulation [18,34]. *f_cl_* is usually measured by different methods (e.g., photographic method, 3D scanning) [37,38,39] or evaluated with empirical equations as a function of the clothing insulation. These equations were derived using typical clothing for moderate and hot environments, like in [37,38] and [39], respectively, and may not be adequate in the case of highly insulating clothing [36]. The only equation for protective clothing against the cold is the one developed in early 2000 in Finland by SubZero project [40,41].

The accuracy of most common formulas for evaluating *f_cl_* from the basic clothing insulation *I_cl_* has been discussed in a paper by Kuklane and Toma [36], who considered a set of measurement carried out on 14 clothing ensembles designed for ambulance personnel covering clothing insulation values from 0.53 to 3.19 clo measured according to ISO 15831 [42]. The clothing area factors were measured with the photographic method [43]. Obtained values were then compared with those predicted by the equations summarized in Table 2.

Additional datasets of *f_cl_* measurements were considered [41] to avoid a one-sided discussion on the topic.

Obtained results [36] demonstrate that all formulas for evaluating the clothing area factor return reliable results for western type and industrial clothing if *I_cl_* ≤ 1.5 clo. For *I_cl_* > 2.0 clo, the equations typically used in the standards (Equations (13), (16) and (17)) and the ones suggested by Smallcombe et al. (Equations (19) and (20)) return an overestimation of the clothing area factor. The calculation accuracy by these equations in the range 1.5 < *I_cl_* ≤ 2 clo may still be acceptable, while equations (Equations (16), (18) and (21)) are highly accurate for *I_cl_* > 2.0 clo. For modern clothing systems based on Western industrial clothing, Equation (22) gives the best fit but requires additional validation on other clothing ensembles. However, as the authors observe [36], a separate question is if and how much different approaches of *f_cl_* calculation affect *IREQ* prediction outcome [18,49].

The second issue discussed in [32] deals with the calculation of the resultant total vapour resistance of clothing *R_e,T,r_* which accounts for the effects of body movements and wind action.

Consistently with ISO 9920 [34,50,51] and ISO 7933 [44], the resultant vapour total clothing resistance can be calculated by multiplying the static *R_e,T_* value given by Equation (23) with a correction factor *CorrE* (as a function of the relative air velocity *v_ar_* and the walking speed *v_w_*) according to Equation (24).

(23) $$R_{e,T}=\frac{I_{T}}{i_{m}L}$$

with *i_m_* = 0.38, *L* = 16.5 K kPa^−1^ and *I_T_* given by Equation (7).

(24) $$R_{eT,r}=R_{e,T}\cdot CorrE$$

with, optionally,

(25) $$CorrE=\exp\left[-0.468\left(v_{\mathrm{ar}}-0.15\right)+0.080{\left(v_{\mathrm{ar}}-0.15\right)}^{2}-0.874v_{w}+0.358v_{w}^{2}\right]$$

or

(26) $$CorrE=0.3-0.5CorrI_{T}+1.2CorrI_{T}^{2}$$

and

(27) $$CorrI_{T}=\frac{I_{T,r}}{I_{T}}$$

and 0.15 m s^−1^ ≤ *v_ar_* ≤ 3.5 m s^−1^, 0 m s^−1^ ≤ *v_w_* ≤ 1.2 m s^−1^.

*CorrI_T_* can be calculated with different equations [34,52] as a function of the total clothing insulation as follows.

Normal or light clothing (e.g., 0.6 clo < I_cl_ < 1.4 clo or 1.2 clo < *I_T_* < 2.0 clo):

(28) $$CorrI_{T}=e^{\left[-0.281\cdot \left(v_{ar}-0.15\right)+0.044\cdot {\left(v_{ar}-0.15\right)}^{2}-0.492v_{w}+0.176v_{w}^{2}\right]}$$

Specialized, insulating, cold weather clothing (e.g *I_T_* > 2 clo):

(29) $$\mathrm{Corr}I_{T}=e^{\left\{\left[-0.0512\cdot \left(v_{ar}-0.4\right)+0.794\cdot 10^{-3}\cdot {\left(v_{ar}-0.4\right)}^{2}-0.0639v_{w}\right]\cdot \;ap^{0.144}\right\}}$$

It is essential to note that more recent algorithms for evaluating *R_e,T,r_* are not available in the literature. Specifically, more recent algorithms [53] focus on local *R_e,T,r_* values to be used in multi-node models [54].

Unlike ISO 9920, the ISO TR 11079: 1993 [49] calculated the resultant vapour resistance by Equation (30):

(30) $$R_{eT,r}=\frac{i_{m}}{L}\left(\frac{I_{a}}{f_{cl}}+I_{cl,r}\right)$$

with

(31) $$I_{cl,r}=\left\{\begin{array}{l} \begin{array}{cccc} 0.90I_{cl} & & if & M<100\;Wm^{-2} \end{array}\\ \begin{array}{cccc} 0.75I_{cl} & & if & M\geq 100\;Wm^{-2} \end{array} \end{array}\right.$$

Fifteen years later, ISO 11079:2007 also applied correction to the basic air boundary layer insulation *I_a_* and introduced the resultant air boundary layer insulation *I_a,r_* and calculates *R_e,T,r_* by means of Equation (32):

(32) $$R_{e,T,r}=\frac{i_{m}}{L}\left(\frac{I_{a,r}}{f_{cl}}+I_{cl,r}\right)=\frac{i_{m}}{L}I_{T,r}$$

Consequently, if Equations (32) and (27) are considered, the correction factor to apply to the static vapour clothing resistance is the same correction factor for the basic total insulation:

(33) $$R_{eT,r}=R_{e,T}\cdot CorrI_{T}$$

This means that the effect of wind and body movements results in the same decrease in clothing vapour resistance *R_e,T_* and total clothing insulation *I_T_* (e.g., CorrE = CorrI_T_). This assumption is inconsistent with measurements analyzed by Havenith et al. [50] who observed a higher reduction in vapour resistance than in total insulation values for three clothing ensembles (1: underwear, trousers, sweater; 2: 1 + cotton coverall; 3: 1 + impermeable coverall).

The approach of the current version of ISO 11079 in dealing with the resultant vapour resistance brings to different values, as observed in previous papers by our team [32,52]. More specifically, *R_eT,r_* values calculated according to Equation (33) are higher than 50% of those calculated according to Equation (24) (air velocity and walking speed values under 2.5 m s^−1^ and 0.6 m s^−1^, respectively) [52]. Such an occurrence could indicate a significant underestimation of latent heat loss through the skin, which has not yet been explored regarding its impact on *IREQ* and *D_lim_* values. In contrast, the effects of various approaches to addressing the resultant vapour resistance have only been examined in the context of predicting heat stress conditions using the PHS model [52,55].

## 3. Methods

The investigation discussed here has been carried out numerically using special software developed in MATLAB (version: 9.13.0) [56], which allows the evaluation of indoor thermal environments [57]. The software section devoted to cold stress assessment via IREQ was specifically designed based on the JAVA Applet available online, corrected by all bugs previously highlighted by our research team [32]. To improve accessibility for users, as performed by other research groups [58], the four MATLAB functions implementing the IREQ model are provided in Appendix B (from Appendix B.1, Appendix B.2, Appendix B.3, Appendix B.4 and Appendix B.5).

Concerning the effect of the clothing area factor on the assessment of cold stress conditions according to ISO 11079, we focused on the equations considered by Kuklane and Toma [36] summarized in Table 2. The analysis consists of two steps:

1. Identification of the equations which return the highest and lowest *f_cl_* values, respectively.
2. Evaluation and comparison of *IREQ* and *D_lim_* values using the equations identified in the previous step, consistently with low strain (neutral) and high strain (minimal) criteria within the range of subjective (metabolic rate and basic clothing insulation) and physical (air temperature, air velocity, relative humidity and mean radiant temperature) variables considered by ISO 11079.

In the second part of this paper, the analysis deals with the effect of the resultant clothing vapour resistance by comparing *IREQ* and *D_lim_* values calculated by Equations (24) and (33), respectively.

The reference conditions for *IREQ*, *D_lim_*, and resultant total evaporative resistance were consistent with those adopted by the figures reported in the ISO 11079 standard [18]. They are summarized in Table 3 and Table 4, respectively.

The effect of clothing area factor has been studied within the following limits of the main parameters as recommended by ISO 11079:

- Air temperature: *t_a_* < 10 °C;
- Air velocity: 0.4 < *v_a_* < 18 m/s;
- Basic clothing insulation, *I_cl_* ≥ 0.5 clo.

The analysis of the effect of the calculation of the resultant total evaporative resistance was limited to *v_a_* = 3.5 m s^−1^ according to the range of applicability of the algorithms recommended by ISO 9920 [34].

## 4. Results and Discussion

### 4.1. The Effect of Clothing Area Factor

#### 4.1.1. Preliminary Observations

**Figure 2:**
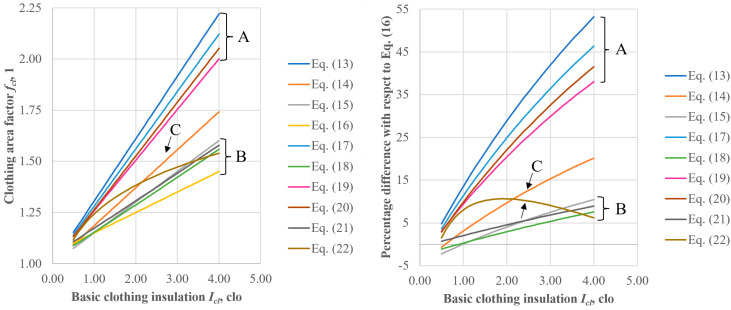
Clothing area factor evaluation according to equations summarized in Table 2 (**left side**) and percentage difference in *f_cl_* calculations by assuming Equation (16) as the reference (**right side**).

In Figure 2, the values of clothing area factor calculated using Equations (13)–(22) are depicted, and the percentage difference with respect to Equation (16) is shown.

Three main groups of formulas can be identified, which provide the highest (A), the lowest (B) and intermediate (C) values of clothing area factor:

- Group A formulas (Equations (13), (17), (19) and (20)) that include ISO 11079 (Equation (13)), ISO 9920 (Equation (17)), and ISO 7933 (Equation (17)) return *f_cl_* values higher than 25% (50%) for *I_cl_* = 2.0 clo (*I_cl_* = 4.0 clo) if compared with Equation (16), which returns the lowest values.
- Group B formulas (Equations (15), (18) and (21)) return *f_cl_* values not higher than 10% if compared with those calculated by Equation (16).
- Group C formulas exhibit intermediate behaviour if compared with Equation (16). More particularly, Equation (14) returns *f_cl_* values not higher than 20%, whereas Equation (22) behaves like group A formulas for *I_cl_* < 1.0 clo, then reaches a maximum of 10.6% for *I_cl_* = 2 clo, and finally overestimates *f_cl_* by 6.2% for *I_cl_* = 4.0 clo.

According to the observation above, the analysis of the effect of the clothing area factor on the calculation of *IREQ* and *D_lim_* values will be focused on the following:

- Equation (13), which returns the highest *f_cl_* values and is adopted by ISO 11079.
- Equation (16), which returns the lowest *f_cl_* values and is chosen as the basis for comparisons.
- Equation (22), which despite requiring further validation, is a better fit for the experimental *f_cl_* values measured on professional modular clothing system for ambulance personnel, which is meant to provide thermal comfort over a wide range of climatic conditions from hot summer days to extremely cold Nordic winters [36].

#### 4.1.2. Effect of Clothing Area Factor on IREQ Values

**Figure 3:**
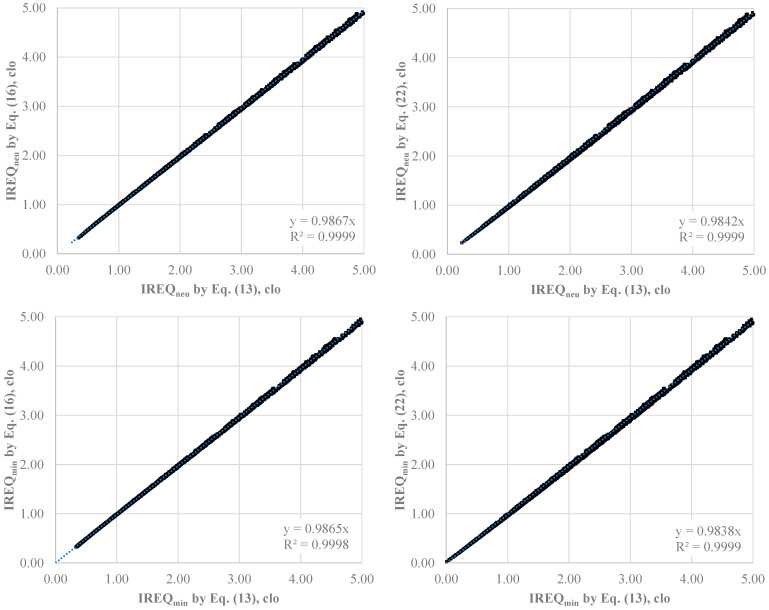
Effect of *f_cl_* formula (Equations (16) and (22)) on the calculation of *IREQ_min_* and *IREQ_neu_*. Input data in Table 3.

Figure 3 shows the effect of *f_cl_* calculations on *IREQ_min_* and *IREQ_neu_* values in the entire range of parameters considered (2744 conditions).

All data quite overlap on the identity line (y = x) with *R^2^* values exceeding 0.999 and slopes a few less than 1: 0.9838 and 0.9842 in the worst cases, that means a negligible underestimation of *IREQ* values of 1.6% in the case of Equations (16) and (22). This phenomenon is quite surprising when we consider that for *I_cl_* = 4.0 clo, Equation (13) yields *f_cl_* values that are 50% higher than those predicted by Equation (16). In contrast, Equations (16) and (22) produce similar results, with a difference not higher than 10% in the range from 0.5 to 4.0 clo.

**Figure 4:**
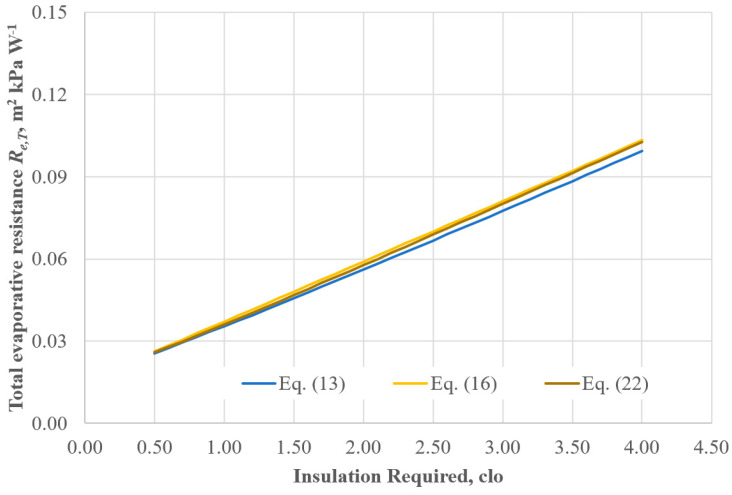
Effect of *f_cl_* formula on the calculation of the total evaporative resistance given by Equation (23) as a function of the IREQ value.

This behaviour is due to the structure of Equation (2), which results in two effects which do not affect both the numerator and denominator of Equation (4):

1. The right side of Equation (2) (dry heat loss) is scarcely affected by the different formulas used for the evaluation of *f_cl_*. This is because the only term at the right side of Equation (2) affected by the clothing area factor is the evaporative heat flow *E* being related to the resultant total evaporative resistance of clothing according to Equations (5) and (32) with I_cl,r_ = IREQ. Now, the total evaporative resistance of clothing and air boundary layer is quite unaffected by the formula adopted for *f_cl_* calculation, as shown in Figure 4.

In fact, according to Equations (13) and (23) for low *IREQ* values, e.g., <1.0 clo, the *f_cl_* variation is not excessive, at 15% at least according to Figure 2, whereas for higher *IREQ* values, the meaningful variation of *f_cl_* values (see Figure 2) related to the different formula is meaningfully softened by the low value of the air boundary layer insulation, *I_a_* = 0.085 m^2^ K W^−1^. As an example, for IREQ = 4 clo, *f_cl_* values given by Equations (16) and (13) are 1.45 and 2.22 (+53%), respectively, while corresponding *R_e,T_* values are almost the same (0.099 and 0.102 m^2^ kPa W^−1^).

**Figure 5:**
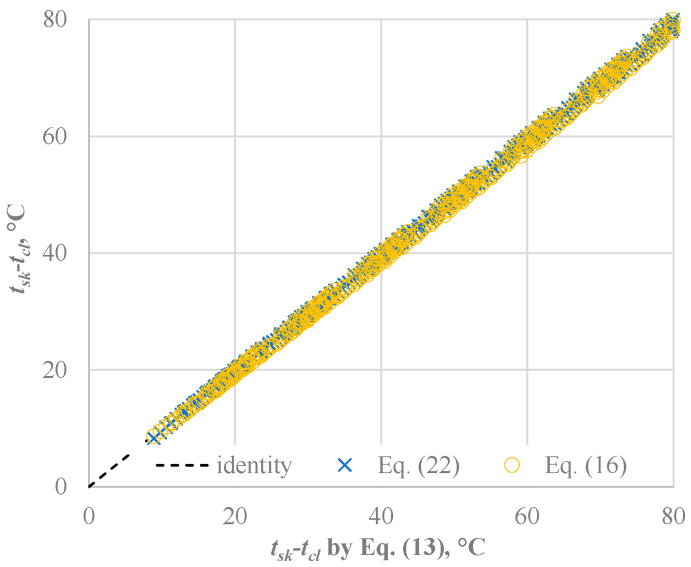
Effect of *f_cl_* formula on the calculation of the temperature difference *t_sk_*-*t_cl_* under thermal neutrality conditions at low strain (*IREQ_neu_*). Input data in Table 3.

2. The clothing surface temperature is scarcely affected by the errors induced by the use of the different formulas for *f_cl_* calculation as confirmed by data shown in Figure 5.

This is the trivial consequence of the presence of the clothing insulation layer of the body surface. More particularly, the value of the difference *t_sk_*-*t_cl_* is close to the difference between the mean skin temperature—which is only affected by the metabolic rate, as shown in Table 1.

#### 4.1.3. Effect of Clothing Area Factor on D_lim_ Values

**Figure 6:**
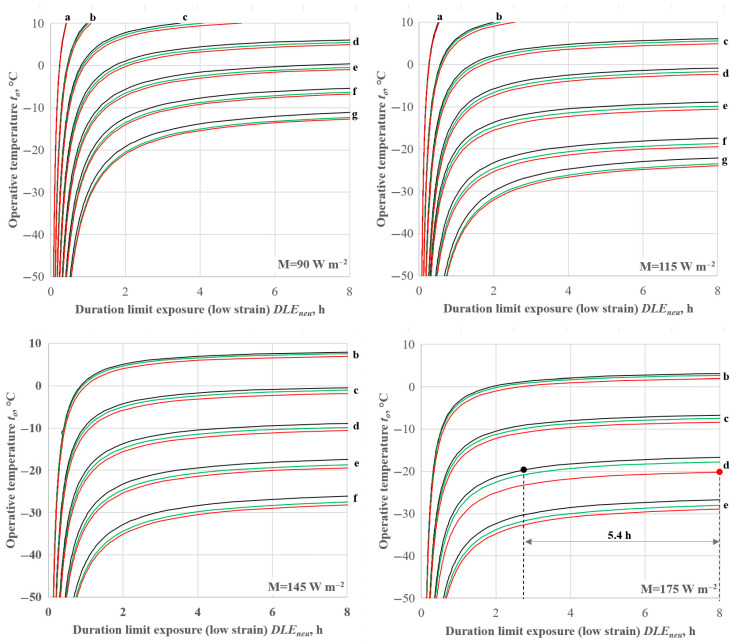
Effect of *f_cl_* formula on the calculation of the duration limit exposure (low strain). Black lines: Equation (13); green lines: Equation (22); red lines: Equation (16). v_a_ = 0.4 m s^−1^; RH = 50%; ap = 8 l m^−2^ s^−1^. (a) I_cl_ = 0.5 clo; (b) I_cl_ = 1.0 clo; (c) I_cl_ = 1.5 clo; (d) I_cl_ = 2.0; (e) I_cl_ = 2.5 clo; (f) I_cl_ = 3.0 clo; (g) I_cl_ = 3.5 clo.

The trends depicted in Figure 6 prove that the *D_lim_* values predicted with Equations (16) and (22) are systematically higher than those calculated with Equation (13), actually adopted by the IREQ model [18].

The reason for this behaviour is that the clothing area factor directly affects the body heat storage rate by increasing the dry heat loss *R + C* and the evaporative term *E*. More specifically, *R*, *C* and *E* increase as the clothing area factor increases according to Equations (3)–(5) and (32). Consequently, the body heat storage rate increases in modulus with the consequent decrease in the *D_lim_* value, according to Equations (34) and (12), respectively.

(34) $$S=M-W-\left(E_{res}+C_{res}+E+R+C\right)$$

**Figure 7:**
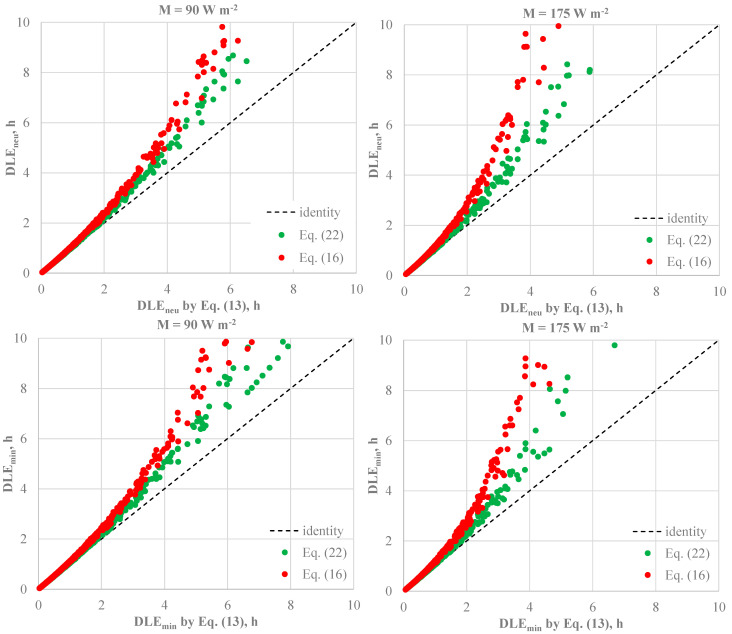
Overestimation of the duration limit exposure predicted by IREQ model by Equations (16) and (22) in the experimental conditions as in Table 3.

From the quantitative perspective, the difference in *D_lim_* values obtained with Equations (16) and (22) is related to the value of the heat storage rate value. More particularly, for high values of the heat storage rate (e.g., at low operative temperature, clothing insulation or low metabolic rate), *D_lim_* is lowest, and even important variations in *f_cl_* do not affect the results. This means that for short-term exposures (e.g., at high values of the heat storage rate *S*), the effect of the clothing area factor is negligible. In contrast, for lower values of the module of the heat storage rate, *D_lim_* is higher. Therefore, the differences are magnified exceeding 5 h in case of Equation (16) as depicted in Figure 6 and Figure 7, which also reports *DLE_min_* values.

Equation (22) shows lower overestimation of *D_lim_* values due to the non-linear behaviour observed in Figure 2. However, for *D_lim_* > 4 h, it returns *D_lim_* values 3 h higher than those calculated with Equation (10).

Based on observed behaviours, it is important to define if Equation (16) or Equation (22) can be adopted by the revision of ISO 11079. On the one hand, Equations (16) and (22) yield more reliable values for the clothing area factor *f_cl_* [36] compared to traditional formulas such as Equation (13), which is currently employed by the IREQ model as specified in the latest ISO 11079 standard [18,59,60]. However, despite their improved accuracy, these equations negatively affect the evaluation of *D_lim_*—particularly during long-term exposure assessments at the verification stage when the resultant clothing insulation *I_cl,r_* falls below the minimum required insulation *IREQ_min_* (Figure 1). This issue arises from their tendency to underestimate the heat storage rate *S*, leading to safety concerns. Therefore, given the limited influence of the *f_cl_* algorithms on IREQ calculations during the design phase—where a clothing ensemble with predefined insulation must be specified—and the absence of physiological validation of the model, we recommend that no changes be made to the current standard.

### 4.2. The Effect of Resultant Vapour Resistance

**Figure 8:**
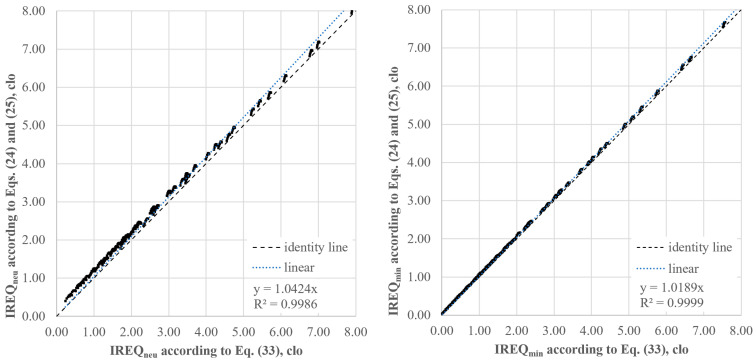
Effect of the resultant vapour resistance calculation on *IREQ_min_* and *IREQ_neu_* under conditions as in Table 4.

Although the *R_e,T,r_* values calculated by Equations (33) and (24) with *CorrE* given by Equation (25) can be meaningfully different—as remarked in a previous investigation by our team [32]—the effect of the calculation of the resultant total vapour resistance on both *IREQ* values is not meaningful, as depicted in Figure 8.

The values of the slopes of the regression lines for *IREQ_neu_* (1.042) and *IREQ_min_* (1.019) demonstrate that the underestimation of the vapour resistance resulting from the application of Equations (24) and (25) [31] and then the higher value of the evaporative heat loss leads to some overestimation *IREQ_neu_* values. More particularly, according to our calculations, this phenomenon is more pronounced in the range of operative temperature from 0 to 10 °C or in the case of high metabolic rate values (175 W m^−2^). For the highest activity level, the effect of the lower vapour resistance is amplified by a higher value of the skin wettedness (as a function of the metabolic rate as in Table 2), resulting in a high evaporative heat loss as quantified by the right side of Equation (5).

In contrast, *IREQ_min_* values are less affected by the value of the *R_e,T,r_* as the slope of the regression line confirms. This is mainly due to the constant value of the skin wettedness (w = 0.06) under high strain conditions (see Table 1).

**Figure 9:**
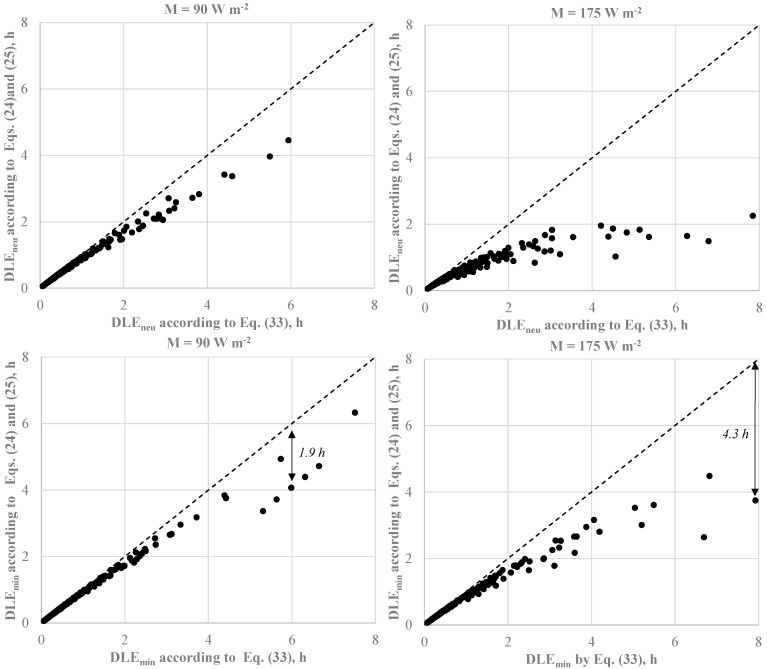
Effect of the resultant vapour resistance calculation on *DLE_min_* and *DLE_neu_* under conditions as in Table 4. Values near arrows represent the maximum difference between DLE values calculated consistently with ISO 11079 (Equation (33)) and ISO 9920 standards (Equations (24) and (25)).

Similarly to what was observed for the effect of the clothing area factor, the impact of the different algorithms for calculating the resultant vapour resistance is more pronounced in cold stress conditions (e.g., when *I_cl,r_* is less than *IREQ*). This is quite surprising if we consider that under cold exposures, the evaporative term in the heat balance equation on the human body is less significant than the dry heat loss (R + C). More particularly, the differences are more pronounced for long-term exposures and high metabolic rates, as depicted in Figure 9, which reveals a systematic underestimation of *D_lim_* by Equation (15). This is the trivial consequence of the higher *R_eT,r_* value returned by Equation (33) resulting in a lower evaporative heat loss and, finally, higher value of the body heat storage *S*.

The underestimation of *D_lim_* values obtained by Equation (33) is more pronounced for exposures over two hours, typical of long-term risks as claimed by ISO 15265 [14,61]. More particularly, for M = 90 W m^−2^ and both physiological criteria reported in Table 1 (low and high strain), Equation (33) overestimated *D_lim_* values by about 2 h (from 1.9 to 2.1 h). For M = 175 W m^−2^, meaningful differences have been observed in the range of *DLE_neu_* values from 2 to 4 h with a maximum overestimation of *DLE_min_* of 5.2 h. Except for a couple of conditions (overestimation of 4.3 h about), *DLE_min_* trend is similar to that observed at lower metabolic rate.

The results mentioned above seem astonishing in cold environments, considering the reduced significance of the evaporative term in the heat balance equation. To understand this apparent inconsistency, a detailed analysis of the contribution of the evaporative term in Equation (34) is necessary. More specifically, according to data in Table 5, for low clothing insulation and operative temperature values (e.g., highest values of the heat body storage rate), even significant variations in the evaporative heat loss (+34.1% for *M* = 90 W m^−2^) do not result in appreciable variations in *S* values.

In fact, the modulus of the body heat storage *S* increases less than 4.2% for *t_o_* ≤ −20 °C. In contrast, for *M* = 175 W m^−2^, the evaporative heat loss variation (+92.5%) due to the lower *R_e,T,r_* value obtained with Equations (24) and (25) results in a 10% increase in *S* even for *t_o_* = −50 °C.

**Figure 10:**
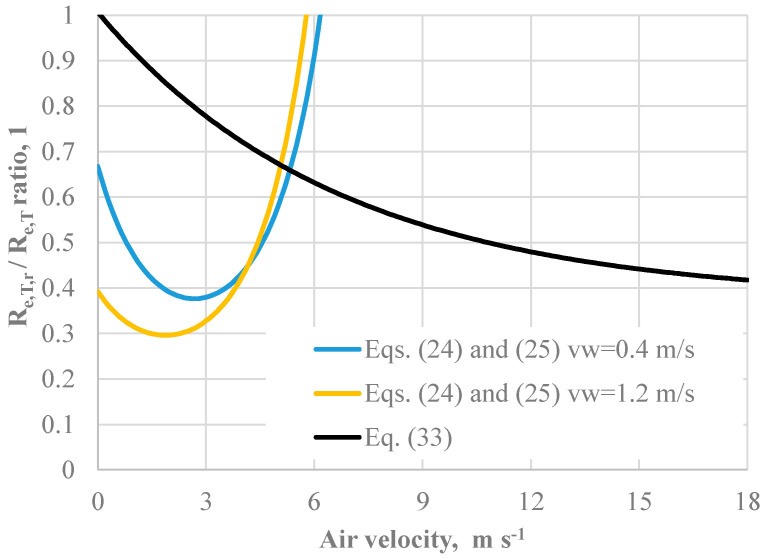
Effect of the air velocity on the ratio between resultant total and total vapour resistance calculated according to Equations (33), (24) and (25).

Based upon the findings above, using Equation (24) with *CorrE* given by Equation (25) in *IREQ* calculations is not recommendable. The reasons are mainly twofold. Above all, Equation (25) has been validated just for v_a_ < 3.5 m s^−1^, while the current version of IREQ works for *v_a_* < 18 m/s. In addition, the application of Equation (25) over its validation range could bring physical inconsistencies. This is mainly because, as shown in Figure 10, it exhibits a minimum and exceeds the unitary value at high air velocity.

Eventually, Equations (24) and (25) could be recommended in the range of air velocity from 0 to 3.5 m s^−1^.

## 5. Conclusions

Despite some limitations, the IREQ model formulated by Holmér in the late 1980s remains the reference method for evaluating global cooling in extreme cold environments. Over the past forty years, the only meaningful modification to the original model consisted of the equations for calculating the resultant clothing insulation to be compared with the *IREQ* value. In contrast, the calculation of the clothing area factor (affecting the dry heat loss and the vapour resistance) and the resultant vapour resistance (affecting the evaporative heat loss) remain unchanged.

Consistently with the issue highlighted in this paper, totally focused on the effects of the new algorithms for the calculation of the clothing area factor *f_cl_* and the resultant vapour resistance, we can schematically state the following:

- Despite the meaningful underestimation of the clothing area factor calculated with the most recent algorithms developed on an experimental basis, the effects on the neutral and minimum *IREQ* values are negligible. Different *f_cl_* formulas return the same *IREQ* values. This phenomenon is only an apparent inconsistency as, under equilibrium conditions (no heat accumulated in the body), the only term affecting the heat balance equation on the human body is the evaporative heat loss that is scarcely affected by *f_cl_*, especially for high clothing insulation values.
- The clothing area factor meaningfully affects the duration limit exposure under long-term risk conditions (*D_lim_* > 2 h and *I_cl,r_* < *IREQ_min_*). More particularly, as *f_cl_* increases, both dry and latent heat losses increase with the consequent increase in the modulus of the body heat storage rate. Consequently, the most recent formula proposed by Kuklane and Toma (lower *f_cl_*, then lower dry heat loss) [36] returns higher *D_lim_* values (up to 5 h) if compared with those calculated consistently with the actual version of ISO 11079 standard.
- The effect of the resultant vapour resistance calculation is similar to that observed in the case of *f_cl_*. *IREQ* values are not meaningfully affected even by meaningful differences in the vapour resistance and the evaporative loss, whereas *D_lim_* values calculated using the actual version of ISO 11079 can be significantly higher than those calculated consistently with the algorithms recommended by ISO 9920 but validated for air velocity values lower than 3.5 m s^−1^.

Based on the results from the present study, we recommend leaving the actual implementation of the IREQ model unchanged unless new experimental evidence demonstrates to modify the evaluation of the resultant clothing thermophysical quantities. If on one side, the most recent studies provide more accurate algorithms for evaluating the clothing area factor as a function of the clothing insulation, then on the other side, their effects on the total and the resultant clothing insulations are unknown. In addition, the algorithms for evaluating the resultant vapour resistance provided by ISO 9920 do not cover the entire range of application of the ISO 11079 in terms of air velocity (18 m s^−1^) and return unacceptable physical inconsistencies when forced over their validation range. Maintaining the current calculation is the most effective approach. Its familiarity ensures easy adoption and understanding among occupational hygienists and environmental ergonomists, who may not grasp intricate changes, especially when the practical effects are negligible.

The results from the present investigation require further validation on an experimental basis, which is difficult to carry out due to the lack of a systematic database of physiological measurements collected in the field or the laboratory (as in the case of extreme hot environments). However, it is important to highlight that the current version of IREQ also lacks experimental validation. So, the obtained results, despite being numerical, provide robust evidence for contributing to its improvement and address further research to focus better on the measurement and evaluation of clothing area factor and resultant vapour resistance for clothing ensembles of interest in cold exposures. This also applies to other indices, such as the Predicted Mean Vote (PMV) and the Predicted Heat Stress (PHS), which use different algorithms for evaluating the clothing area factor and the resultant vapour resistance if compared with those recommended by ISO 9920.

In the immediate future, the development of this research will proceed along two parallel paths. Firstly, new experimental measurements will be conducted on mobile mannequins to determine the optimal method for calculating dynamic vapour resistance in severe cold environments. Additionally, significant efforts will be made to address the validation and refinement of the IREQ model. Specifically, physiological measurements will be performed under both laboratory and field conditions. Furthermore, the IREQ model will be validated by comparing its predicted values for heat storage rate and duration limit exposure with those obtained from the most advanced thermo-physiological models, such as Fiala Ergonsim, THERMODE, and JOS-3.

## Figures and Tables

**Table 1:** Suggested physiological criteria for the determination of *IREQ* and *D_lim_* [18].

Quantity	Low Strain	High Strain
IREQ	Neutral (IREQ_neu_)	Minimal (IREQ_min_)
t_sk_	35.7–0.0285·M	33.34–0.0354·M
w	w = 0.001·M	0.06
D_lim_	Short	Long
Q_lim_ (kJ m^−2^)	144	144

**Table 2:** Some clothing area factor formulas considered by Kuklane and Toma [36]. *I_cl_* is expressed in m^2^ K W^−1^.

Equation	Equation Number	References
1.0 + 1.97 *I*_cl_	(13)	[18,44]
1.0 + 1.197 *I*_cl_	(14)	[45]
1.0 + 0.97 *I*_cl_	(15)	[46]
1.05 + 0.645 *I*_cl_	(16)	[40,41,47]
1.0 + 1.81 *I*_cl_	(17)	[34]
1.0 + 0.85 *I*_T_	(18)	[40,41]
1.01 +1.599 *I*_cl_	(19)	[48]
1.0 + 1.697 *I*_cl_	(20)	[48]
1.04 + 0.85 *I*_T_	(21)	[36]
1.657 *I*_cl_^0.1546^	(22)	[36]

**Table 3:** Experimental conditions for the evaluation of the effect of *f_cl_* formulas on *IREQ* and *D_lim_* calculations. ^(^*^)^ Air temperature and mean radiant temperature are assumed to be equal; ^(^**^)^ *I_cl_* values of 0.5, 3.0, and 3.5 clo have not been considered in simulations with M = 175 W m^−2^.

Variable	Units	Values	Number of Conditions
IREQ calculations			
t_a_	°C	from −50 to 10 (step 10 °C)	7
RH	%	50	1
t_r_	°C	from −50 to 10 (step 10 °C)	7
v_a_	m s^−1^	0.5, 1.0, 1.5, 2, 5, 10, 18	7
M	W m^−2^	70, 90, 115, 145, 175, 200, 230, 260	8
ap	L m^−2^ s^−1^	8	1
Number of simulations			2744
D_lim_ calculations			
t_a_ ^(^*^)^	°C	from −50 to 10 (step 10 °C)	7
RH	%	50	1
v_a_	m s^−1^	0.5, 1.0, 1.5, 2.0, 5.0, 10, 18	7
M	W m^−2^	90, 115, 145, 175	4
I_cl_	clo	0.5 ^(^**^)^, 1.0, 1.5, 2.0, 2.5, 3.0 ^(^**^)^, 3.5 ^(^**^)^	7
ap	L s^−1^ m^−2^	8	1
Number of simulations			1225

**Table 4:** Experimental conditions for the evaluation of the effect of the calculation of the resultant total evaporative resistance on *IREQ* and *D_lim_* calculations. ^(^*^)^ Air temperature and mean radiant temperature are assumed to be equal; ^(^**^)^ *I_cl_* values of 0.5, 3.0, and 3.5 clo have not been considered in simulations with M = 175 W m^−2^.

Variable	Units	Values	Number of Conditions
t_a_^(^*^)^	°C	from −50 to 10 (step 10 °C)	7
RH	%	50	1
v_a_	m s^−1^	from 0.5 to 3.5 (step 0.5 m s^−1^)	7
M	W m^−2^	90, 175	2
I_cl_	clo	0.5 ^(^**^)^, 1.0, 1.5, 2.0, 2.5, 3.0 ^(^**^)^, 3.5 ^(^**^)^	7
ap	L m^−2^ s^−1^	8	1
Number of simulations			539

**Table 5:** Effect of the calculation of the resultant vapour resistance on the evaporative heat flow at the skin *E* and the body heat storage rate *S* as a function of the air temperature, clothing insulation and metabolic rate under low strain conditions (Table 1). v_a_ = 0.5 m s^−1^; RH = 50%, ap = 8 L s^−1^ m^−2^. Positive values of the body heat storage rate have not been reported.

t_o_ (°C)	E (W m^−2^)		Δ (%)	S (W m^−2^)		Δ (%)	E (W m^−2^)		Δ (%)	S (W m^−2^)		Δ (%)
	Equation (33)	Equations (24) and (25)		Equation (33)	Equations (24) and (25)		Equation (33)	Equations (24) and (25)		Equation (33)	Equations (24) and (25)	
	M = 90 W m^−2^						M = 175 W m^−2^					
I_cl_ = 1.0 clo												
−50	13.9	18.7	34.1	−337	−341	1.4	24.9	48.0	92.5	−284	−307	8
−40	13.9	18.6	34.1	−288	−293	1.6	24.9	47.9	92.5	−232	−255	10
−30	13.9	18.6	34.1	−239	−244	2.0	24.8	47.8	92.5	−180	−203	13
−20	13.8	18.5	34.1	−190	−195	2.5	24.6	47.4	92.5	−128	−151	18
−10	13.6	18.2	34.1	−141	−146	3.3	24.2	46.6	92.5	−76	−98.2	30
0	13.1	17.5	34.1	−92	−97	4.8	23.2	44.7	92.5	−23	−44.3	94
10	12.2	16.4	34.1	−42	−47	9.9	21.5	41.3	92.5	-	-	-
I_cl_ = 1.5 clo												
−50	10.5	14.1	34.1	−236	−239	1.5	18.9	36.3	92.5	−178	−196	10
−40	10.5	14.1	34.1	−199	−202	1.8	18.8	36.3	92.5	−139	−156	13
−30	10.5	14.1	34.1	−162	−165	2.2	18.8	36.2	92.5	−99	−116	18
−20	10.4	14.0	34.1	−125	−128	2.9	18.6	35.9	92.5	−59	−76.6	29
−10	10.3	13.8	34.1	−88	−91	4.0	18.3	35.2	92.5	−20	−36.5	87
0	9.9	13.3	34.1	−50	−54	6.7	17.6	33.8	92.5	-	-	-
10	9.3	12.4	34.1	−12	−15	25.7	16.2	31.3	92.5	-	-	-
I_cl_ = 2.0 clo												
−50	8.4	11.3	34.1	−174	−177	1.7	15.1	29.1	92.5	−113	−127	12
−40	8.4	11.3	34.1	−144	−147	2.0	15.1	29.1	92.5	−81	−95	17
−30	8.4	11.3	34.1	−114	−117	2.5	15.1	29.0	92.5	−49	−63	28
−20	8.4	11.2	34.1	−84	−87	3.4	15.0	28.8	92.5	−17	−31	81
−10	8.2	11.0	34.1	−54	−57	5.2	14.7	28.3	92.5	-	-	-
0	7.9	10.7	34.1	−24	−27	11.2	14.1	27.1	92.5	-	-	-
10	7.4	10.0	34.1	-	-	-	13.0	25.1	92.5	-	-	-
I_cl_ = 2.5 clo												
−50	7.0	9.4	34.1	−132	−134	1.8	12.6	24.2	92.5	−69	−81	17
−40	7.0	9.4	34.1	−107	−109	2.2	12.6	24.2	92.5	−42	−54	28
−30	7.0	9.4	34.1	−82	−84	2.9	12.5	24.2	92.5	−15	−27	76
−20	7.0	9.3	34.1	−57	−59	4.2	12.5	24.0	92.5	-	-	-
−10	6.9	9.2	34.1	−32	−34	7.3	12.2	23.5	92.5	-	-	-
0	6.6	8.9	34.1	−7	−9	33.5	11.7	22.6	92.5	-	-	-
10	6.2	8.3	34.1	-	-	-	10.9	20.9	92.5	-	-	-

## Data Availability

The original contributions presented in this study are included in the article. Further inquiries can be directed to the corresponding author(s).

## Symbols

The following symbols are used in this manuscript:

*A*_Du_	Dubois body surface area, m^2^
*a*_p_	air permeability of outer fabric, L s^−1^ m^−2^
*A*_r_	Effective radiating area of a body, m^2^
*C*	Convective heat flow, W m^−2^
*C*_res_	Respiratory convective heat flow, W m^−2^
*CorrE*	Correction factor for total vapour resistance, ND
*CorrI_T_*	Correction factor for total insulation, ND
*DLE*_min_	Duration limit exposure consistent with high strain criteria, h
*DLE*_neu_	Duration limit exposure consistent with low strain criteria, h
*D*_lim_	Duration limit exposure, h
*E*	Evaporative heat flow at the skin, W m^−2^
*E*_res_	Respiratory evaporative heat flow, W m^−2^
*f*_cl_	Clothing area factor, ND
*h*_c_	Convective heat transfer coefficient, W m^−2^ K^−1^
*h*_r_	Radiative heat transfer coefficient, W m^−2^ K^−1^
*I*_a_	Static boundary layer thermal insulation, m^2^ K W^−1^ or clo
*I_a,r_*	Resultant boundary layer thermal insulation, m^2^ K W^−1^ or clo
*I*_cl_	Basic clothing insulation, m^2^ K W^−1^ or clo
*I*_cl,r_	Resultant clothing insulation, m^2^ K W^−1^ or clo
*i*_m_	Moisture permeability index, ND
*IREQ*	Required clothing insulation, m^2^ K W^−1^ or clo
*IREQ_min_*	Minimal required clothing insulation, m^2^ K W^−1^ or clo
*IREQ_neu_*	Neutral required clothing insulation, m^2^ K W^−1^ or clo
*I_T_*	Basic total insulation, m^2^ K W^−1^ or clo
*I_T,r_*	Resultant total insulation, m^2^ K W^−1^ or clo
*K*	Conductive heat flow, W m^−2^
*L*	Lewis relation, K kPa^−1^
*M*	Metabolic rate, W m^−2^ or met
*p*_a_	Water vapour partial pressure, kPa
*p*_ex_	Saturated water vapour pressure at expired air temperature, kPa
*p*_sk,s_	Saturated water vapour pressure at skin temperature, kPa
*R*	Radiative heat flow, W m^−2^
*R_e,T_*	Total evaporative resistance of clothing and boundary air layer, m^2^ kPa W^−1^
*R*_e,T,r_	Resultant evaporative resistance of clothing and boundary air layer, m^2^ kPa W^−1^
*RH*	Relative Humidity, %
*Q*_lim_	Limit body heat loss, kJ m^−2^ or W h m^−2^
*S*	Body heat storage rate, W m^−2^
*t*_a_	Air temperature, °C
*t*_cl_	Clothing surface temperature, °C
*t*_ex_	Expired air temperature, °C
*t*_o_	Operative temperature, °C
*t*_r_	Mean radiant temperature, °C
*t*_sk_	Mean skin temperature, °C
*v*_a_	Air velocity, m s^−1^
*v*_ar_	Relative air velocity, m s^−1^
*v*_w_	Walking speed, m s^−1^
*W*	Effective mechanical power, ND
*w*	Skin wettedness, ND

## Greek Symbols

The following Greek symbols are used in this manuscript:

*ε*_cl_	Emissivity of clothing surface, ND
*σ*	Stefan–Boltzmann constant, W m^−2^ K^−4^

## Abbreviations

The following abbreviations are used in this manuscript:

PHS	Predicted Heat Strain
PMV	Predicted Mean Vote
SOBANE	Screening, OBservation, Analysis, Expertise

## Appendix A

The presence of a fourth-order term (see Equation (3b)) in the non-linear system of Equation (A1), which has to be solved to determine the IREQ value, necessitates the use of an iterative procedure.

(A1) $$\left\{\begin{array}{l} R+C=M-W-E_{\mathrm{res}}-C_{\mathrm{res}}-E\\ IREQ=\frac{t_{sk}-t_{cl}}{R+C} \end{array}\right.$$

where [18,35,47,49]:

(A2) $$C_{res}=0.0014M\left(t_{ex}-t_{a}\right)$$

(A3) $$E_{res}=0.0173M\left(p_{ex}-p_{a}\right)$$

(A4) $$t_{ex}=29+0.2t_{a}$$

(A5) $$p_{ex}=p_{s}(t_{ex})$$

with

p_a_ water vapour partial pressure, kPa;

p_ex_ saturated water vapour pressure at expired air temperature, kPa;

t_ex_ expired air temperature, °C.

This method is well established in the field of the ergonomics of physical environments [18,35,44,47,62]. It is also implemented by the algorithms of the ASHRAE 55 standard [63], as well as by most common open-source software [58,64]. The fundamental steps of this procedure are briefly outlined in this section, while specific software implementation details are described in Appendix B.

1. Since the four microclimatic quantities (air temperature, air velocity, relative humidity, and mean radiant temperature) and the metabolic rate are known, the right side of the first equation in system (A1) is a known value, except for the evaporative heat flow. With this in mind, we arbitrarily choose a trial value for IREQ (e.g., IREQ = 0.5)
2. The resultant evaporative resistance can be evaluated using Equation (27) with the assumed value for IREQ (I_cl,r_ = IREQ). Consequently, the evaporation rate at the skin surface, given by Equation (5), can be calculated by assuming the skin wettedness values provided by Table 1. At this point, the right-hand side of Equation (2) becomes a known quantity, allowing the calculation of the clothing temperature with the following equation (see Equation (A1)):

(A6)
$$t_{cl}=t_{sk}-IREQ\left(M-W-E_{\mathrm{res}}-C_{\mathrm{res}}-E\right)$$

3. The value of *t*_cl_ obtained from Equation (A6) enables the calculation of the radiative heat transfer coefficient *h_r_* and the convective heat transfer coefficient *h_c_*, as given by Equations (A7) and (A8), respectively. Consequently, the convective and radiative heat fluxes can be determined using Equations (3a) and (3b).

(A7)
$$h_{r}=\frac{R}{t_{cl}-t_{r}}=\varepsilon_{cl}\sigma \frac{A_{r}}{A_{Du}}\frac{\left[{\left(t_{cl}+273\right)}^{4}-{\left(t_{r}+273\right)}^{4}\right]}{t_{cl}-t_{r}}$$

(A8)
$$h_{c}=\frac{1}{I_{a,r}}-h_{r}$$

4. The final step involves comparing the left-hand side of Equation (2), calculated as described in bullet point 3, with the right-hand side, calculated as outlined in bullet point 2. Specifically,
  (a) If the left-hand side exceeds the right, the trial IREQ value is increased by a small, arbitrary increment (see the adjustment factor in codes reported in the Appendix B).
  (b) If the right-hand side exceeds the left, the trial IREQ value is decreased by a similarly small amount.

In both cases, the calculations in bullet points 2 and 3 are repeated iteratively until the values converge.

## Appendix B

This appendix provides the Matlab functions that implement the IREQ model in accordance with ISO 11079 [18], along with an example Matlab script detailing input and output data.

### Appendix B.1. Matlab Function for the Calculation of IREQ Neutral

function [I_neu ] = IREQ_neu(ta, tr, va, RH, M, W, im)

%Matlab function for the evaluation of IREQ value: low strain criterion (neutral)

%Developed by InEqualiTIES team @ DII (University of Naples Federico II,

%Naples Italy) and DIIN (University of Salerno, Italy) 2025

%INPUT DATA

%ta,  air temperature, °C

%tr,  mean radiant temperature, °C

%va,  air velocity, m/s

%RH,  relative humidity, %

%M,   metabolic rate, W/m^2^

%W,   effective mechanical power, W/m^2^

%Icl, basic clothing insulation, m^2^K/W

%im,  moisture permeability index, 1

%OUTPUT DATA

%I_neu, IREQ neutral, m^2^K/W

%Instructions begin here%

 walk = 0.0052*(M − 58);

if walk >= 0.7

     walk = 0.7;

end

tsk = 35.7 − 0.0285*M;

wetness = 0.001*M;

tex = 29 + 0.2*ta;

pex = 0.1333*exp(18.6686 − 4030.183/(tex + 235));

psks = 0.1333*exp(18.6686 − 4030.183/(tsk + 235));

if ta >= 0

     pa = (RH/100)*0.1333*exp(18.6686 − 4030.183/(ta + 235));

else

     pa = (RH/100)*0.6105*exp(21.875*ta/(265.5 + ta));

end

 Iar = 0.092*exp(−0.15*va − 0.22*walk) − 0.0045;

IREQ = 0.5;

aradu = 0.77;

factor = 0.5;

balance = 1;

 while abs(balance) > 0.01

     fcl = 1 + 1.97*IREQ;

     ReT = (0.06/im)*(Iar/fcl + IREQ);

     E = wetness*(psks − pa)/ReT;

     Hres = 0.0173*M*(pex − pa) + 0.0014*M*(tex − ta);

     tcl = tsk − IREQ*(M − W − E − Hres);

     hr = 0.0000000567*0.97*aradu*((273 + tcl)^4 − (273 + tr)^4)/(tcl − tr);

     R = fcl*hr*(tcl − tr);

     hc = 1/Iar − hr;

     C = fcl*hc*(tcl − ta);

     balance = M − W − E − Hres − R − C;

     if (balance > 0)

             IREQ = IREQ − factor;

             factor = factor/2;

     else

             IREQ = IREQ + factor;

     end

end

I_neu = (tsk − tcl)/(R + C);

End

### Appendix B.2. Matlab Function for the Calculation of IREQ Minimal

function [I_min] = IREQ_min(ta, tr, va, RH, M, W, im)

%Matlab function for the evaluation of IREQ value: high strain criterion (minimum)

%Developed by InEqualiTIES team @ DII (University of Naples Federico II,

%Naples Italy) and DIIN (University of Salerno, Italy) 2025

%INPUT DATA

%ta,  air temperature, °C

%tr,  mean radiant temperature, °C

%va,  air velocity, m/s

%RH,  relative humidity, %

%M,   metabolic rate, W/m^2^

%W,   effective mechanical power, W/m^2^

%Icl, basic clothing insulation, m^2^K/W

%im,  moisture permeability index, 1

%OUTPUT DATA

%I_neu, IREQ minimum, m^2^K/W

%Instructions begin here%

walk = 0.0052*(M − 58);

if walk >= 0.7

     walk = 0.7;

end

tsk = 33.34 − 0.0354*M;

wetness = 0.06;

tex = 29 + 0.2*ta;

pex = 0.1333*exp(18.6686 − 4030.183/(tex + 235));

psks = 0.1333*exp(18.6686 − 4030.183/(tsk + 235));

if ta >= 0

     pa = (RH/100)*0.1333*exp(18.6686 − 4030.183/(ta + 235));

else

     pa = (RH/100)*0.6105*exp(21.875*ta/(265.5 + ta));

end

Iar = 0.092*exp(−0.15*va − 0.22*walk) − 0.0045;

IREQ = 0.5;

aradu = 0.77;

factor = 0.5;

balance = 1;

while abs(balance) > 0.01

      fcl = 1 + 1.97*IREQ;%fcl secondo la 11079

      ReT = (0.06/im)*(Iar/fcl + IREQ);

      E = wetness*(psks − pa)/ReT;

      Hres = 0.0173*M*(pex − pa) + 0.0014*M*(tex − ta);

      tcl = tsk − IREQ*(M − W − E − Hres);

      hr = 0.0000000567*0.97*aradu*((273 + tcl)^4 − (273 + tr)^4)/(tcl − tr);

      R = fcl*hr*(tcl − tr);

      hc = 1/Iar − hr;

      C = fcl*hc*(tcl − ta);

      balance = M − W − E − Hres − R − C;

      if (balance > 0)

             IREQ = IREQ − factor;

             factor = factor/2;

      else

             IREQ = IREQ + factor;

      end

end

I_min = (tsk − tcl)/(R + C);

end

### Appendix B.3. Matlab Function for the Calculation of DLE Neutral

function [D_neu] = DLE_neu(ta, tr, va, RH, M, W, Icl, im, ap)

%Matlab function for the evaluation of the duration limit exposure via IREQ

%model: low strain criterion (neutral)

%Developed by InEqualiTIES team @ DII (University of Naples Federico II,

%Naples Italy) and DIIN (University of Salerno, Italy) 2025

%INPUT DATA

%ta,  air temperature, °C

%tr,  mean radiant temperature, °C

%va,  air velocity, m/s

%RH,  relative humidity, %

%M,   metabolic rate, W/m^2^

%W,   effective mechanical power, W/m^2^

%Icl, basic clothing insulation, m^2^K/W

%im,  moisture permeability index, 1

%ap,  air permeability of outer fabric, L/m^2^s

%OUTPUT DATA

%D_neu duration limited exposure (neutral), h

%Instructions begin here%

walk = 0.0052*(M − 58);

if walk >= 1.2

     walk = 1.2;

end

tsk = 35.7 − 0.0285*M;

wetness = 0.001*M;

tex = 29 + 0.2*ta;

pex = 0.1333*exp(18.6686 − 4030.183/(tex + 235));

psks = 0.1333*exp(18.6686 − 4030.183/(tsk + 235));

if ta >= 0

     pa = (RH/100)*0.1333*exp(18.6686 − 4030.183/(ta + 235));

else

     pa = (RH/100)*0.6105*exp(21.875*ta/(265.5 + ta));

end

Iar = 0.092*exp(−0.15*va − 0.22*walk) − 0.0045;

tcl = ta;

S = −40;

aradu = 0.77;

factor = 100;

Iclr = Icl;

balance = 1;

while abs(balance) > 0.0001

      fcl = 1 + 1.97*Iclr;

Iclr = ((Icl + 0.085/fcl)*(0.54*exp(0.075*log(ap) − 0.15*va − 0.22*walk) − 0.06*log(ap) + 0.5)) − Iar/fcl;

      ReT = (0.06/im)*(Iar/fcl + Iclr);

      E = wetness*(psks − pa)/ReT;

      Hres = 0.0173*M*(pex − pa) + 0.0014*M*(tex − ta);

      tcl = tsk − Iclr*(M − W − E − Hres − S);

      hr = 0.0000000567*0.97*aradu*((273 + tcl)^4 − (273 + tr)^4)/(tcl − tr);

      R = fcl*hr*(tcl − tr);

      hc = 1/Iar − hr;

      C = fcl*hc*(tcl − ta);

      balance = M − W − E − Hres − R − C − S;

      if (balance > 0)

             S = S + factor;

             factor = factor/2;

      else

             S = S − factor;

      end

end

if S < 0

      D_neu = −40/S;

else

      D_neu = 9,999,999;

end

end

### Appendix B.4. Matlab Function for the Calculation of DLE Minimal

function [D_min] = DLE_min(ta, tr, va, RH, M, W, Icl, im, ap)

%Matlab function for the evaluation of the duration limit exposure via IREQ

%model: high strain criterion (minimum)

%Developed by InEqualiTIES team @ DII (University of Naples Federico II,

%Naples Italy) and DIIN (University of Salerno, Italy) 2025

%INPUT DATA

%ta,  air temperature, °C

%tr,  mean radiant temperature, °C

%va,  air velocity, m/s

%RH,  relative humidity, %

%M,   metabolic rate, W/m^2^

%W,   effective mechanical power, W/m2

%Icl, basic clothing insulation, m^2^K/W

%im,  moisture permeability index, 1

%ap,  air permeability of outer fabric, L/m^2^s

 %OUTPUT DATA

%D_min duration limited exposure (minimum), h

%Instructions begin here%

walk = 0.0052*(M − 58);

if walk >= 1.2

     walk = 1.2;

end

tsk = 33.34 − 0.0354*M;

wetness = 0.06;

tex = 29 + 0.2*ta;

pex = 0.1333*exp(18.6686 − 4030.183/(tex + 235));

psks = 0.1333*exp(18.6686 − 4030.183/(tsk + 235));

if ta >= 0

     pa = (RH/100)*0.1333*exp(18.6686 − 4030.183/(ta + 235));

else

             pa = (RH/100)*0.6105*exp(21.875*ta/(265.5 + ta));

end

Iar = 0.092*exp(−0.15*va − 0.22*walk) − 0.0045;

tcl = ta;

S = −40;

aradu = 0.77;

factor = 100;

Iclr = Icl;

balance = 1;

 while abs(balance) > 0.0001

      fcl = 1 + 1.97*Iclr;

      Iclr = ((Icl + 0.085/fcl)*(0.54*exp(0.075*log(ap) − 0.15*va − 0.22*walk) − 0.06*log(ap) + 0.5)) − Iar/fcl;

      ReT = (0.06/im)*(Iar/fcl + Iclr);

      E = wetness*(psks − pa)/ReT;

      Hres = 0.0173*M*(pex − pa) + 0.0014*M*(tex − ta);

      tcl = tsk − Iclr*(M − W − E − Hres − S);

      hr = 0.0000000567*0.97*aradu*((273 + tcl)^4 − (273 + tr)^4)/(tcl-tr);

      hc = 1/Iar − hr;

      R = fcl*hr*(tcl − tr);

      C = fcl*hc*(tcl − ta);

      balance = M − W − E − Hres − R − C − S;

      if (balance > 0)

             S = S + factor;

             factor = factor/2;

      else

             S = S − factor;

      end

end

 if S < 0

   D_min = −40/S;

else

   D_min = 99,999;

end

end

### Appendix B.5. Example of Matlab Script for Calculating IREQ Values

%Air temperature value, °C

ta = 5;

%Mean radiant temperature value, °C

tr = 5;

%Air velocity value, m/s

va = 1;

%Relative humidity value, %

RH = 50;

%Metabolic rate value, W/m^2^

M = 90;

%Effective mechanical power, W/m^2^

W = 0;

%Moisture permeability index value, 1

im = 0.38;

%Calculation of IREQneu and IREQmin in m^2^K/W

IREQneu = IREQ_neu(ta, tr, va, RH, M, W, im);

IREQmin = IREQ_min(ta, tr, va, RH, M, W, im);
